# Supplementary material for: Relaxin-like Gonad-Stimulating Peptides in Asteroidea
Source: Biomolecules. 2023 Apr 30;13(5):781. doi: 10.3390/biom13050781 (PMC10216564; doi:10.3390/biom13050781)
Supplement: Supplementary file 1 [file biomolecules-13-00781-s001.zip › biomolecules-2264315-supplementary.pdf]

Table S1. The Sequence Read Archives (SRAs) used in the analysis of RGP orthologs. RGP homologs were identified by searching the sequence data bases of SRA and identified by BLAST analysis against Trinity (https://github.com/trinityrnaseq) assembled contig sequences with the transcriptome sequence data.

Orders	Species	SRAs	Accession numbers of RGP and RGPR
Valvatida	*Patiria* (*Asterina*) *pectinifera*	SRR8627925	AB496611, LC027938, LC027939
			LC757025
		SRR8627925	LC567883, LC757026
		SRR8627926	
	Patiria miniate	SRR2454338	LC057686
	Parvulaster exigua	SRR10389879	
	Pateriella regularius	ERR995409	
	Asteropsis carnifera	SRR2843236	
	Acanthaster cf solaris (*planci*)	SRR8613694	LC033566, LOC110989162
	Cernardoa semiregularis	SRR16157127	LC066682
	Glabraster antarctica	SRR2844627	
Paxillosida	*Astropecten duplicatus*	SRR28443238	
	Astropecten latespinosus	DRR179610	
	Astropecten scoparius	SRR12736189	LC131032
	Luidia quinaria	SRR16157128	
	Luidia clathrate	SRR1139195	
	Luidia senegalensis	SRR3217895	
Forciplatida	*Asterias amurensis*	SRR1139201	LC040882
	Asterias rubens	SRR1642063	KT601728
	Aphelasterias japonica	SRR16157129	LC104980
	Marthasterias glacialis	ERR6436374	
	Piaster ochraceus	SRR2846074	
	Coscinaster acutispina	—	LC131033
	Labidiaster annulatus	SRR2845324	
Velatida	*Pteraster tesselatus*	SRR2846094	
Spinulosida	*Henricia leviuscua*	SRR2844835	
	Echinaster spinulosus	SRR1139455	LC131034
